# p53 induces distinct epigenetic states at its direct target promoters

**DOI:** 10.1186/1471-2164-9-486

**Published:** 2008-10-15

**Authors:** Lukas Vrba, Damian J Junk, Petr Novak, Bernard W Futscher

**Affiliations:** 1 Arizona Cancer Center, the University of Arizona, Tucson, AZ 85724, USA; 2 Cancer Biology Graduate Interdisciplinary Program, the University of Arizona, Tucson, AZ 85724, USA; 3 Department of Pharmacology and Toxicology, College of Pharmacy, the University of Arizona, Tucson, AZ 85724, USA; 4 Biology Centre ASCR, v.v.i., Institute of Plant Molecular Biology, Ceske Budejovice, 37005, Czech Republic

## Abstract

**Background:**

The tumor suppressor protein p53 is a transcription factor that is mutated in many cancers. Regulation of gene expression by binding of wild-type p53 to its target sites is accompanied by changes in epigenetic marks like histone acetylation. We studied DNA binding and epigenetic changes induced by wild-type and mutant p53 in non-malignant hTERT-immortalized human mammary epithelial cells overexpressing either wild-type p53 or one of four p53 mutants (R175H, R249S, R273H and R280K) on a wild-type p53 background.

**Results:**

Using chromatin immunoprecipitation coupled to a 13,000 human promoter microarray, we found that wild-type p53 bound 197 promoters on the microarray including known and novel p53 targets. Of these p53 targets only 20% showed a concomitant increase in histone acetylation, which was linked to increased gene expression, while 80% of targets showed no changes in histone acetylation. We did not observe any decreases in histone acetylation in genes directly bound by wild-type p53. DNA binding in samples expressing mutant p53 was reduced over 95% relative to wild-type p53 and very few changes in histone acetylation and no changes in DNA methylation were observed in mutant p53 expressing samples.

**Conclusion:**

We conclude that wild-type p53 induces transcription of target genes by binding to DNA and differential induction of histone acetylation at target promoters. Several new wild-type p53 target genes, including *DGKZ*, *FBXO22 *and *GDF9*, were found. DNA binding of wild-type p53 is highly compromised if mutant p53 is present due to interaction of both p53 forms resulting in no direct effect on epigenetic marks.

## Background

The wild-type (wt) p53 protein acts as a transcription factor that responds to a variety of stress stimuli that pose a threat to normal cells. In response to various genotoxic stresses, wt p53 binds specific sequence elements in the promoters of its target genes such as *p21*, *MDM2*, *PUMA*, and *PCNA *[1-8]. Binding of wt p53 results in the recruitment of the co-activator p300/CBP and subsequent acetylation of promoter associated histones H3 and H4 [1,2,6-8]. Binding of this activation complex and increased histone acetylation was linked to increased expression of these genes. Activation of wt p53 also causes repression of a subset of its target genes such as *MAP4*, *AFP*, *BCL2*, *survivin*, and various cell cycle regulatory genes [9-13]. Chromatin immunoprecipitation studies have shown binding of wt p53 to the promoter regions of some of these genes [9-11]. This binding was associated with the recruitment and binding of the co-repressors SIN3A and HDAC1, and subsequent decreases in histone H3 and H4 acetylation. Interestingly, gene repression in response to wt p53 in some cases also requires an interaction with other DNA binding molecules such as SP1 and the NF-Y complex [9,10]. Therefore, wt p53 binds the promoters of its target genes and recruits either co-activators or co-repressors that modify histone H3 and H4 acetylation levels resulting in hyper-acetylated histones and increased gene expression [1,2,6,7] or hypo-acetylated histones and decreased gene expression [9-11]. Since wt p53 transcriptional activity is associated with DNA-binding, a variety of computational and whole genome chromatin immunoprecipitation techniques have been utilized to identify direct p53 target genes [14-20].

p53 protein consists of distinct functional domains [21,22]. The N-terminal region is involved in transactivation and the core of the protein forms a DNA binding domain that interacts specifically with DNA target sequences. The domain in the C-terminal region enables oligomerization of p53 protein, which binds to DNA as a tetramer. Since wt p53 is a critical tumor suppressor gene, it is inactivated in many human cancers [23]. Interestingly, inactivation of wt p53 often occurs as single point mutation in the core DNA binding domain, suggesting the importance of DNA binding for the tumor suppressor function of wt p53. These mutations of the p53 DNA binding domain can be divided into two groups [24]. Structure mutations are amino acid residue changes that cause perturbation of the structure of the DNA binding surface of p53 protein. Most common p53 structure mutations appear in codons R175, G245, R249, and R282 [24]. Contact mutations are replacements of amino acid residues that normally make direct contact with the DNA. Common p53 contact mutants include codons R248, R273, and R280 [24]. In 15–30% of breast cancers, inactivation of p53 occurs by mutation, making this the most common genetic defect related to a single gene [25-28]. Microarray analyses have demonstrated that accumulation of mutant (mt) p53 indeed causes changes in the expression of a variety of genes, and indicates it still may affect gene transcription [29-33]. Chromatin immunoprecipitation experiments have shown that mt p53 can bind the promoter regions of genes *in vivo*; however, efforts to identify mt p53 specific DNA-binding response elements have failed [32-34].

In the present study, we investigated DNA-binding and epigenetic modifications in response to induced levels of wt and mt/wt p53 using non-malignant hTERT immortalized human mammary epithelial cells (HME1). To simulate a wt p53 response, we used transient adenoviral infection of wt p53. To determine the effect of mt p53 on wt p53 we created four stable cell lines over-expressing the R175H, R249S, R273H, and R280K p53 mutants in a wt p53 background [35]. These p53 mutants were chosen because they represent some of the most common p53 mutations forming together over 13% of all p53 mutation cases identified in breast cancer [36]. Both methods of p53 overexpression resulted in comparable levels of mt/wt or wt p53 protein. To identify p53 binding sites in these scenarios and p53's effect on the epigenetic state, we conducted chromatin immunoprecipitation coupled to microarray hybridization that analyzed 13,000 human gene promoters.

Our study is unique from previously published results in the fact that it analyzes not only binding of wt but also the binding of mt/wt p53 on a genome wide scale. Previous studies analyzed either only wt p53 binding on a genome wide scale [15,16,18-20] or binding of selected p53 mutants to a few selected p53 target gene promoters [8]. To our knowledge, our study is also the first to identify the changes in histone acetylation induced by wt or mt/wt p53 on a genome wide scale. Moreover, our model examines this role of p53 in the context of non-malignant mammary epithelial cells, in contrast to the malignantly transformed colon, lung and osteosarcoma cells used in previous studies. Taken together, this mt/wt p53 model provides new insights into p53 dysfunction during an early point in human mammary carcinogenesis when mt p53 mutation coexists with wt p53.

Our results show that wt p53 binds a multitude of promoter sequences causing increases in histone H3 and H4 acetylation. Some of these promoter sequences belong to novel, previously undescribed, p53 target genes. This DNA-binding and increase in histone acetylation in response to wt p53 is associated with increases in gene expression. We did not find any direct wt p53 binding associated with decreases in histone acetylation or gene expression. In the mt/wt p53 state over 95 % of p53 specific DNA binding was inhibited. The loss in p53 binding resulted in very little change in histone H3 and H4 acetylation and no changes in DNA methylation. The results of our investigation demonstrate a lack of wt p53 repressive binding and mt p53 DNA-binding as a whole. Our data suggests that wt p53 DNA-binding is associated with increased histone acetylation and gene expression of a multitude of target genes, including several new wt p53 targets.

## Results

### Cell line treatments

Direct binding of p53 to target promoters and the effect of over-expression of wt and mt p53 on the epigenetic state of promoters was studied in a non-malignant hTERT immortalized breast epithelial cell line, HME1. Wt p53 is toxic when overexpressed in these cells; therefore the attempt to prepare cell lines stably overexpressing wt p53 was not successful. Thus, transient overexpression of wt p53 from an adenoviral vector was used to induce a wt p53 response and the level of p53 expression was consistent with a physiological stress response. The p53 mutants R175H, R249S, R273H and R280K were stably overexpressed in HME1 cells containing endogenous wt p53 to analyze the effect that mt p53 had on wt p53's function as a transcription factor. We have shown previously that wt p53 accumulated in response to mt p53 overexpression in these cells [35], and the accumulation of wt p53 likely occurred due to stabilization as a result of its interaction with mt p53 [35]. The combined level of wt and mt p53 protein in these cell lines was comparable to the level of wt p53 in cells overexpressing wt p53 from the adenoviral vector [35]. The cell lines created and used within the study described in this paper are summarized in Table 1.

**Table 1:** Cell line models used in this study

Name	mutated codon	mutation	amino acid change	vector	promoter	overexpression	p53 phenotype
HME1	None	none	none	none	none	none	wt parental

Ad5WT	None	none	none	Adenovirus	CMV	transient (24h)	wt accumulated

R175H	175	GCG→GCA	Arg→His	Lentivirus	CMV	stable	mt/wt accumulated

R249S	249	AGG→AGC	Arg→Ser	Lentivirus	CMV	stable	mt/wt accumulated

R273H	273	GCG→GCA	Arg→His	Lentivirus	CMV	stable	mt/wt accumulated

R280K	280	GAG→GAA	Arg→Lys	Lentivirus	CMV	stable	mt/wt accumulated

The table displays the names of samples used within this study, mutated codon position and nucleotide and amino acid change in p53 if any, vector and promoter used for p53 variant overexpression, type of overexpression and p53 phenotype of the studied cell lines.

### Genome-wide p53 promoter binding

First, we identified p53 binding to DNA targets using a ChIP on chip method. The DO-1 monoclonal antibody, which is targeted against amino acids 21–25 of p53 protein and recognizes both the wt and mt p53 proteins, was used in our study. DNA from chromatin immunoprecipitated by the DO-1 antibody was labeled and hybridized to a 13,000 human gene promoter microarray. The probes on this microarray represent 13,000 human gene promoters and are PCR products that cover the regions 700 base pairs upstream to 200 base pairs downstream of transcription start [37]. Input DNA, i.e. DNA from unimmunoprecipitated chromatin, was co-hybridized as a reference. DO-1 immunoprecipitation from MDA-MB-157 cell line that has p53 null phenotype and does not express p53 protein detectable by western blot (data not shown) was used as the other reference.

**Figure 1:**
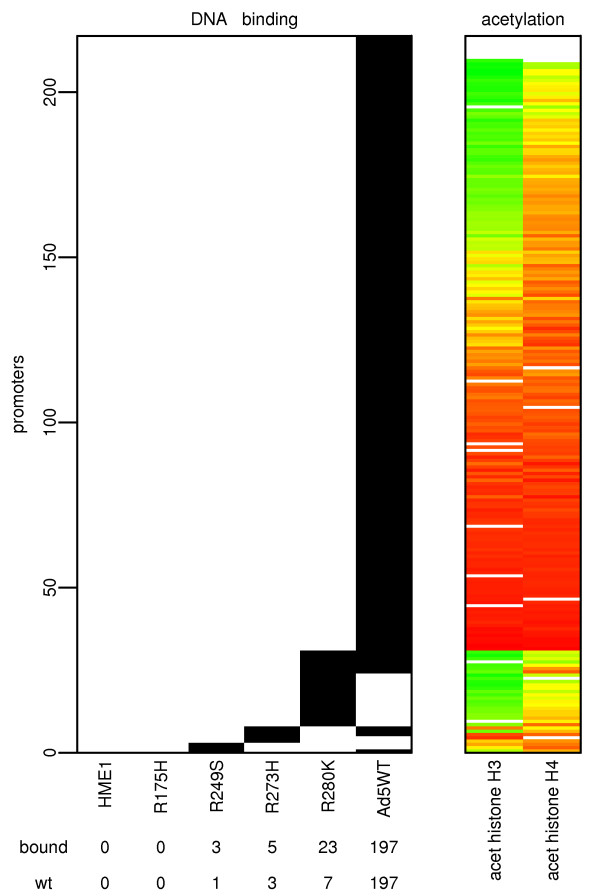
**Numbers of p53 bound promoters in studied cell lines**. The number of promoters bound by p53 in the studied cell lines and their average acetylation detected by chromatin immunoprecipitation and hybridization to 13,000 human gene promoter microarray. The left panel displays number of promoters bound by p53 and their overlap between samples. Data are from three independent experiments including dye swap, therefore six hybridizations per sample. Promoters that have shown enrichment over both input and p53 null reference (MDA-MB-157) samples at p-value 0.01 or lower were considered bound. The cell line labels, numbers of promoters bound in each cell line, and numbers of promoters bound in each cell line that overlap with those bound in Ad5WT are displayed below the x-axis. The numbers of promoters are represented by the y-axis. The black boxes are promoters bound by p53 in a particular cell line. Only promoters bound by p53 in at least one cell line are shown. The total list of p53 bound promoters is available as additional file 1. The right panel displays the average acetylation of histones H3 and H4 in promoters displayed on the left panel. The level of acetylation is repreresented by a heatmap. Green color reflects higher acetylation, red color lower acetylation. The block of only highly acetylated promoters corresponding to promoters bound by R280K mt p53 is clearly visible.

The parental HME1 cell line with basal levels of wt p53 showed no significant p53 binding to any of the promoters on the microarray (Fig 1A). In contrast, HME1 cells with transiently overexpressed wt p53 (Ad5WT) exhibited significant binding to a variety of different gene promoters. Wt p53 in these cells was found to bind to 197 promoters, which represents nearly 2% of the analyzed promoters (Fig 1A). The bound promoters included many known p53 transcriptional targets such as *PLK3*, *FAS*, *APAF1*, *C12orf5*, *PCNA*, *TP53INP1*, *DDB2*, *MASPIN*, *GDF15 *and *PIG11*. In addition to known p53 targets there was a group of gene promoters with significant binding that had not been previously described as p53 targets. These genes, which include *FBXO22*, *DGKZ*, *MGC4771*, *PCM1*, *GDF9*, *DPAGT1*, *SKI*, *SYK*, *OVOL1 *and *PLXNB3*, were identified as potential novel p53 targets. The total list of wt p53 bound promoters is provided in additional file 1

Expression of mt p53 protein on the wt p53 HME1 background inhibited DNA binding. Despite the high level of wt p53 protein in cells overexpressing mt p53, the presence of mt p53 led to a greater than 95% reduction in p53 binding to its targets. The cell line with overexpressed mt R175H showed no promoter binding. p53 in the remaining three cell lines overexpressing R249S, R273H and R280K mt p53 bound to only 3, 5 and 23 promoters on the microarray, respectively (Fig 1A). The promoters bound in R273H and R280K significantly overlap with promoters bound in the wt p53 only expressing cell line (Ad5WT). All overlaps between p53 binding in mt/wt samples and p53 binding in Ad5WT sample with respective probabilities that overlaps are just random were 1 promoter for R249S (p-val = 5.6 × 10^-2^), 3 promoters for R273H (p-val = 6.6 × 10^-5^) and 7 promoters for R280K (p-val = 1.5 × 10^-7^) (Fig 1A). The list of promoters bound by mt/wt p53 is shown for each mutant in additional file 1. A substantial number of promoters bound by the mutants were not detected as bound in the Ad5WT cells (Fig. 1A). These data suggest that the presence of mt p53 inhibits the binding of wt p53 to its targets, and possibly allows for binding to non-target sites. Further study revealed that p53 in the R280K mt binds only promoters with a high level of histone acetylation (Fig 1B). Our data indicate that wt p53 at basal levels does not bind its target sites and that the presence of a mt p53 can block elevated levels of wt p53 from binding target promoters.

### Genome-wide assessment of epigenetic modifications induced by p53 binding

**Figure 2:**
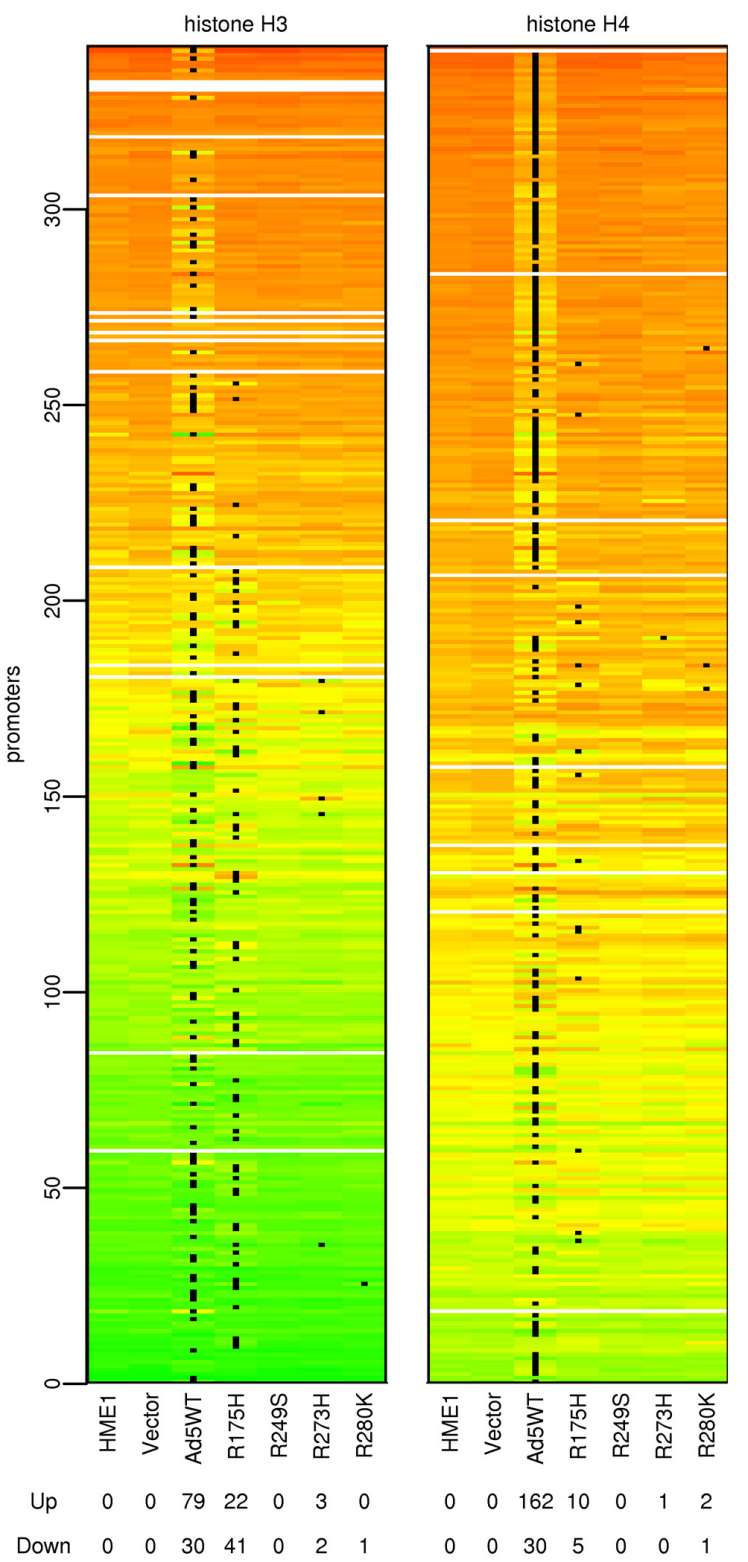
**Changes in acetylation of histones H3 and H4 in studied cell lines**. Heatmaps show histone acetylation status of promoters that demonstrate significant changes from control (parental and vector only transformed) cell lines in any sample detected by chromatin immunoprecipitation and hybridization to 13,000 human gene promoter microarray. Promoters were sorted in the y-axis direction by decreasing average acetylation. Green color reflects high acetylation, red color low acetylation. The average acetylation from three independent experiments is shown. Cell line labels are displayed below the x-axis. The two bottom lines display the number of promoters that were significantly (p-val ≤ 0.05) more or less acetylated than in the control cell lines, and are shown by black dots within the figure. Acetylation of histone H3 is shown on the left and acetylation of histone H4 on the right. The total list of differentially acetylated promoters is available as additional file 2.

Since a number of p53 targets become epigenetically silenced in cancer, we tested whether p53 overexpression invokes epigenetic changes such as altered acetylation of histones H3 and H4 and methylation of DNA. For histone acetylation the chromatin was immunoprecipitated using antibodies against acetylated histones H3 or H4 as two independent marks of changes in chromatin. DNA from immunoprecipitated samples was labeled and hybridized to the 13,000 human gene promoter microarray using input DNA as a reference. The changes in histone acetylation relative to parental and vector only transformed cell lines were calculated. Most significant changes in histone acetylation occurred in response to overexpression of wt p53. Histone H3 became significantly more acetylated in 79 promoters and significantly less acetylated in 30 promoters in this sample (Fig 2A). Acetylation of histone H4 increased in 162 promoters and decreased in 30 promoters in response to wt p53 (Fig 2B). The total list of differentially acetylated promoters is available as additional file 2.

A different situation was observed in the mt p53 expressing cell lines. The only mt cell line with a significantly changed histone H3 acetylation pattern was R175H with 22 promoters with increased acetylation and 41 promoters with decreased acetylation. The histone H3 acetylation in the remaining three mt p53 cell lines was similar to the parental cell line. The R249S showed no significant changes, R273H had 3 promoters with increased and 2 promoters with decreased acetylation, and R280K had 1 promoter with decreased acetylation (Fig 2A). Similar results were obtained for acetylation of histone H4 (Fig 2B). Interestingly, the mt R280K that demonstrated the most DNA binding of all mutants, had nearly no effect on histone acetylation, and bound only to promoters that were already highly acetylated (Fig 1B).

In order to determine if mt p53 alters DNA methylation state, DNA methylation was analyzed using two microarray platforms. One platform was a 6,800 element CpG island microarray. This DNA microarray contains ds DNA probes that cover CpG rich regions dispersed throughout the human genome, including single copy regions as well as alu and satellite repeat elements [38]. The other platform was the 13,000 human gene promoter microarray used for the p53 binding and histone acetylation study. DNA samples from each of the four mt p53, parental HME1 and vector only transformed cell lines were analyzed using a McrBC digestion technique and hybridization to the CpG island microarray. No significant changes in DNA methylation state in response to long-term mt p53 overexpression were found (data not shown). To verify these observations, DNA from parental HME1, vector only, and the R175H mt cells which demonstrated the most changes in histone acetylation, was immunoprecipitated with a 5-methylcytosine specific antibody. Labeled DNA was hybridized to the 13,000 human gene promoter microarray against input DNA as a reference. No significant changes in DNA methylation were found (data not shown).

Our data suggest that overexpression of wt p53 affected histone acetylation of a multitude of gene promoters. The expression of mt p53 forms did not have an effect on histone acetylation, with the exception of the R175H mt. This p53 mutant, however, does not bind DNA at all, so the observed changes were likely an indirect effect of expression of R175H mt p53 protein in the cell. Despite changes in acetylation of histones in R175H mt, we did not find significant changes in DNA methylation in any mutant expressing cell lines. The methylation of DNA in the cell line overexpressing wt p53, which is harvested a short time after infection (24 h), was not determined because earlier results suggest that DNA methylation does not change in such a short time frame [39].

### Changes in acetylation in response to wt p53 binding

**Figure 3:**
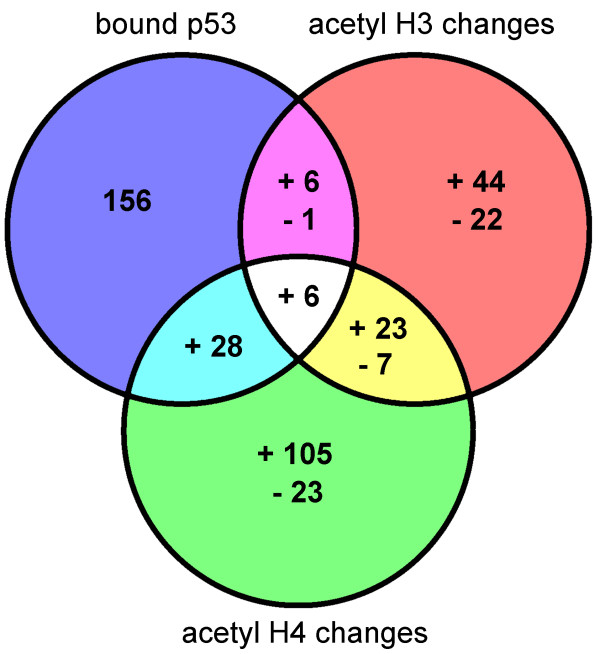
**Overlap between wt p53 DNA binding and changes in histone acetylation**. The Venn diagram shows the overlap between promoters bound by wt p53 and promoters with significant changes in acetylation of histones H3 and H4 in response to wt p53 overexpression. The number of promoters with increased acetylation are marked with a (+) sign and those with decreased acetylation with a (-) sign.

The only cell line that showed a significant number of p53 bound promoters and changes in histone acetylation was HME1 overexpressing wt p53 (Ad5WT). Therefore, we studied whether there is an overlap between the promoters that were bound by wt p53 and those whose acetylation has changed in these cells. These data are summarized in figure 3. Approximately 20 % (41 out of 197, Fig 3) of the promoters bound by wt p53 had significant changes in acetylation of histone H3 or H4. This overlap is highly significant, i.e. there exists a correlation between promoters bound by p53 and those with changed acetylation, the p-value of this overlap is lower than 2.2 × 10^-16^. Therefore we conclude that a correlation exists between wt p53 DNA binding and changes in histone acetylation. The majority of these promoters (40 out of 41) had increased histone acetylation, consistent with wt p53 as a transcriptional activator.

### p53 binding and expression analysis using real-time PCR

**Figure 4:**
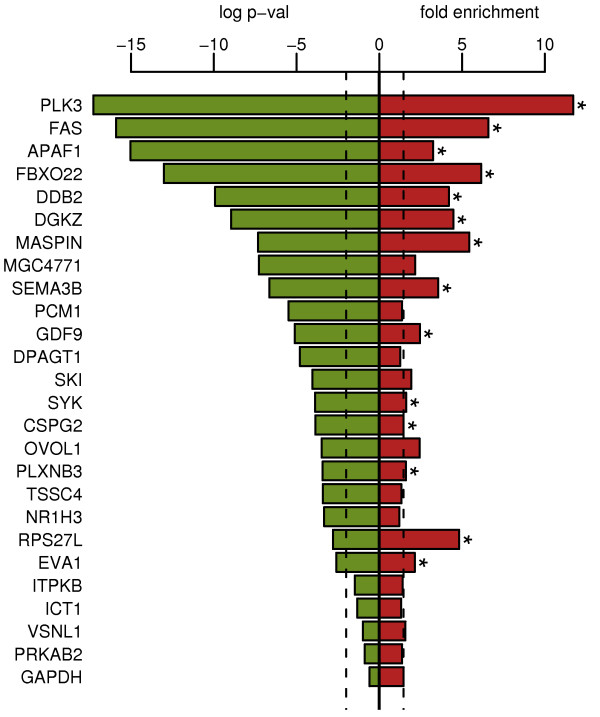
**Real-time PCR verifies wt p53 binding detected by microarray analysis**. The left panel displays the significance level of p53 binding to selected promoters according to the microarray data. The x-axis displays log(p-value) of enrichment over input. The vertical dashed line shows the p-value 0.01 which was used as a limit. The right panel depicts fold enrichment of p53 bound promoters as detected by chromatin immunoprecipitation and real-time PCR. The x-axis displays fold enrichment of immunoprecipitated sample over input. The vertical dashed line shows the level of the negative control gene *GAPDH*, which is not enriched. The genes that had significant increases in expression are marked with an asterisk.

To verify the microarray p53 binding data, real-time PCR was performed to validate and determine the level of enrichment of selected promoter sequences in immunoprecipitated samples. We tested the promoters of 25 genes and *GAPDH *as a negative control. The greatest enrichment over input was demonstrated by the promoter of the gene *PLK3 *(11.7 fold, Fig 4). This is consistent with microarray binding data where this promoter reached the lowest p-value (5.24 × 10^-18^) for enrichment over input. No positive promoters were confirmed by real-time PCR when the p-value was > 0.01 (Fig 4 left panel). This data suggests that the p-value used to decide whether a promoter is bound by p53 or not is appropriate.

**Figure 5:**
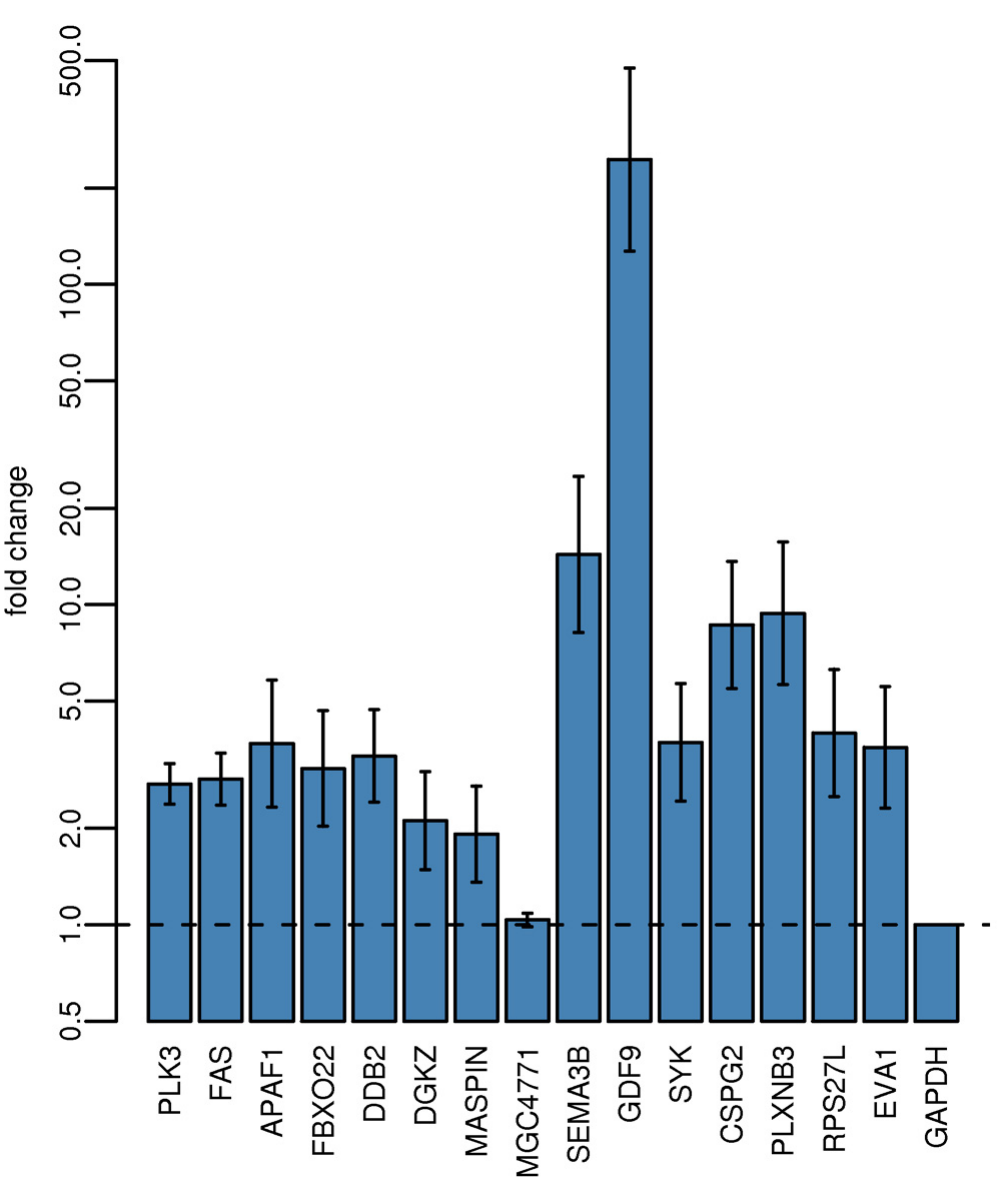
**Real-time RT-PCR demonstrates increased expression of genes bound wt p53**. The bar graph depicts real-time RT-PCR detection of changes in expression in response to wt p53 overexpression. 15 selected genes whose promoters were found to be bound by wt p53 are displayed with *GAPDH *as a negative control. The y-axis is in logarithmic scale and displays fold change in expression relative to control cell line. The horizontal dashed line shows the relative level of expression in the control sample. The data were normalized relative to *GAPDH*. The error bars display the standard error of three replicates.

Because p53 binding is likely to change the expression of its target genes, the expression of selected genes was studied using real-time RT-PCR. Most of the tested genes shared an increase in expression from 2 fold (*MASPIN*) up to 246 fold (*GDF9*) over control in response to wt p53 overexpression (Fig 5). p53 binding data, changes in histone acetylation of histones H3 and H4 and changes in expression for 13 selected genes bound by wt p53 according to the ChIP on chip analysis are summarized in Table 2. These genes include previously known p53 targets such as *APAF1*, *FAS*, *PLK3 *and *MASPIN *and several genes that were not previously described as p53 transcriptional targets. The new p53 targets include *FBXO22*, *DGKZ*, *GDF9*, *SYK*, and *PLXNB3*.

**Table 2:** Summary of data for select known and novel wt p53 targets

gene name	binding p-value	enrichment microarray	enrichment real-time PCR	H3	H4	expression change
PLK3	5.24E-18	2.3	11.7	1.7*	2.0*	2.7

FAS	1.26E-16	1.9	6.6	1.1	1.1	2.9

APAF1	9.04E-16	1.5	3.2	1.4*	1.3	3.7

FBXO22*	9.64E-14	1.6	6.1	1.3*	1.2	3.1

DDB2	1.19E-10	1.3	4.2	1.1	1.0	3.4

DGKZ*	1.12E-09	1.5	4.5	1.8*	1.7*	2.1

MASPIN	4.81E-08	1.2	5.4	1.3*	1.1	1.9

SEMA3B	2.31E-07	1.2	3.6	1.4	1.3	14.3

GDF9*	7.69E-06	1.3	2.4	1.2*	1.2	246.1

SYK*	1.33E-04	1.2	1.6	1.1	1.0	3.7

CSPG2	1.38E-04	1.1	1.5	1.5*	1.3*	8.6

PLXNB3*	3.81E-04	1.2	1.6	1.4*	1.1	9.4

RPS27L	1.58E-03	1.2	4.8	1.4	1.3*	4.0

Novel direct p53 target genes are marked with an asterisk. The p-value column displays the p-value of enrichment of p53 ChIP sample over input detected by the microarray. The next two columns display fold enrichment of ChIP sample over input detected by microarray hybridization or real-time PCR, respectively. The histone H3 and H4 columns display fold changes in histone acetylation relative to the control cell lines. Significant changes (p-val ≤ 0.05) in histone acetylation are marked with an asterisk. The expression column displays fold increase in expression, detected by real-time RT-PCR, relative to control.

Our data suggest that epigenetic changes, such as acetylation of histones H3 and H4 are induced in response to overexpression of wt p53 and some p53 mutants in the breast epithelial cell model system, but DNA methylation is not affected by the presence of mt p53. This study also identified several new, previously undescribed, transcriptional targets of wt p53.

## Discussion

We describe in this report the DNA binding capacity, histone acetylation changes, and DNA methylation changes in response to wt p53 accumulation alone and wt p53 accumulation that occurs with overexpression of mt p53. We had previously demonstrated that overexpression of exogenous mt p53 by lentiviral stable insertion caused a concomitant increase in endogenous wt p53 in HME1 cells [35]. This accumulation of endogenous wt p53 occurs as a result of interaction with the more stable mt p53 protein. The wt and mt p53 molecules form heterotetramers and the average ratio of wt and mt p53 in these heterotetramers was 1:1 [35]. Therefore, this report describes the binding and transactivation effects of wt p53 alone, as well as mt/wt p53 heterotetramers, which would simulate a heterozygous mt/wt state.

Mutations of the p53 DNA binding domain can be divided into two groups [24]. Structure mutations are amino acid residue changes that cause perturbation of the structure of the DNA binding surface of p53 protein. Contact mutations are replacements of amino acid residues that normally make direct contact with the DNA. According to this classification, of the 4 mt p53 investigated in our study, R175H and R249S are structure mutations whereas R273H and R280K are contact mutations.

The structure mt R175H did not show any DNA binding on our promoter microarray, and we conclude that the altered conformation of this mt p53 protein likely affects the conformation of the entire mt/wt heterotetramer and completely compromises its binding to DNA. The changes in histone acetylation we observed in this mt are therefore most likely the secondary effects of overexpression of mt p53 protein and not a direct effect of mt p53 binding to DNA. The mt protein with changed structure possibly forms new interactions with other proteins. Effect of R175H mt could be also caused by preventing the binding of wt p53 to sites that are occupied under normal conditions. However, the relative occupancy of p53 target sites in untreated HME1 cells is so low, if any, that it was not detected by our ChIP on chip analysis as statistically significant.

The structure mt R249S bound only 3 promoters (only 1 of these promoters was bound in Ad5WT sample) and did not invoke any changes in histone acetylation. The contact mt R273H bound 5 promoters (3 of them were bound in Ad5WT) and induced nearly no changes in histone acetylation. The R249S and R273H mutants severely compromised p53 DNA binding, only allowing 1–2% of wt p53 binding. This data likely explains why we did not detect many changes in histone acetylation in these two cell lines.

The contact mt R280K retained the highest DNA binding of the four mutants tested in our study. Interestingly, it bound only to promoters with a high basal level of histone acetylation and many of these targets appear to be non-specific as they were not bound by wt p53. This observation suggests that this p53 mt has altered DNA binding activity and prefers regions of highly acetylated histones, i.e. actively transcribed chromatin. This binding is probably not specific to any particular target DNA sequence. Additionally, binding of this mutant did not cause many changes in histone acetylation, possibly because of the fact that it bound only promoters that were already highly acetylated.

Of the four mutants tested in our study, the structure mutants R175H and R249S had more compromised DNA binding than the contact mutants R273H and R280K. This could be the result of more severe changes in protein structure. However, the level of p53 protein in cells overexpressing the structure mutants R175H and R249S was about 2/3 that seen in the R273H and R280K [35]. So, lower levels of p53 protein causing lower DNA binding can not be completely excluded. Nonetheless, our experimental data show that the binding of p53 in the four mutant expressing cell lines was highly compromised in comparison with binding of wt p53.

We detected significant increases and decreases in histone acetylation in response to wt p53 overexpression. However, consistent with wt p53 function as a transcriptional activator, we found mostly increases in histone acetylation overlapping p53 binding. This overlap between promoters that were bound by wt p53 and those that showed significant increases in acetylation of either histone H3 or H4 was highly significant. The overlapping group, however, formed only about 20% of the bound promoters. The remaining 80% of the bound promoters showed no significant changes in acetylation, however, most of these promoters were already highly acetylated in untreated cells so that a further increase due to p53 binding was impossible or undetectable as significant.

The high proportion of promoters with increase in histone acetylation without p53 binding (Fig 3) may be due to the limited region covered by microarray probes. p53 can bind much further upstream than the region covered by the probes on the promoter microarray (700 bp upstream to 200 bp downstream of transcription start). For example *p21*, one of the best known p53 responsive genes, has two p53 binding sites located 2.3 and 1.4 kbp upstream of transcription start [40]. Therefore, the microarray used in this study is able to detect changes in acetylation in the transcriptional start regions of such genes, but, if p53 binding occurs several kbp up or down stream, this microarray could not detect this event. Finally, many genes with changed acetylation without detected p53 binding may also be secondary targets, downstream on the p53 regulated response pathway.

With one exception, we have not detected any significant decreases in histone acetylation for the promoters bound by p53. These results are not in complete agreement with other published in some other studies [9-11] where a decrease in histone acetylation was observed at some p53 target genes. This apparent discrepancy could be due to differences in models employed (malignant versus non-malignant cells, cell types analyzed), the fact that not all transcriptional down regulation is linked to histone deacetylation, and the different analytical tools employed in the individual studies. For example, using a DNA sequencing strategy, Wei et al [19] observed that the p53 binding sites of genes that are transcriptionally activated tend to cluster around the transcriptional start of the gene. By extension p53 binding sites in targets of transrepression may have a tendency to occur at regions removed from promoter regions, and therefore would not be detected by the human gene promoter microarray we employed. Alternatively, p53 transrepression and associated histone deacetylation may occur through protein-protein interactions that do not involve direct binding of p53 to DNA [10], and therefore would not be effectively detected using ChIP-based approaches.

Our study revealed several potential new p53 target genes. We have tested the expression of selected candidates in response to p53 overexpression by real-time RT-PCR. According to our data, the genes *SYK*, *GDF9*, *DGKZ*, and *FBXO22 *are direct p53 targets demonstrated by ChIP on chip analysis and realtime RT-PCR analysis, which have not been described before. There are numerous studies suggesting that SYK (spleen tyrosine kinase) acts as a tumor suppressor in breast cancer and human melanoma as its overexpression inhibits invasiveness of both types of tumors [41-44]. GDF9 (growth differentiation factor 9) has been shown to reduce the invasiveness of breast cancer cells [45]. Suggesting that *SYK *and *GDF9 *are tumor suppressor genes is consistent with our observation that they are direct p53 transcriptional targets. DGKZ (diacylglycerol kinase zeta) binds pRB and overexpression of DGKZ in pRB-null fibroblasts reconstitutes a cell cycle arrest induced by gamma-irradiation [46]. Participation in cell cycle arrest is consistent with being a p53 target. FBXO22 (F-box protein 22) belongs to the family of f-box proteins [47] which are one of the four subunits of ubiquitin protein ligases. F-box proteins thus comprise the specificity of substrate for ubiquitination. The FBXO22 could, therefore, be involved in degradation or inactivation of specific proteins in response to p53 induction. Therefore we conclude that the novel p53 targets have functions consistent with the tumor suppressive activity of their regulator wt p53.

Overall, we observed binding of induced wt p53 to almost two hundred gene promoters including known and novel wt p53 targets. Binding of wt p53 in about 20% of cases was accompanied by increased histone acetylation followed by increased expression. We did not observe any significant decreases in histone acetylation directly driven by wt p53 binding. The mt p53 highly compromised p53 binding to DNA when expressed on a wt p53 background. Therefore, there were no direct changes in histone acetylation and no changes in DNA methylation observed in these cell line models.

## Conclusion

Wt p53 when overexpressed from adenoviral vector in HME1 cells bound 197 promoters of the human gene promoter microarray. p53 binding resulted in statistically significant increases in histone acetylation of either histone H3 or histone H4 or both for 40 of these promoters. We observed no decreases in histone acetylation for genes bound by p53, so we conclude that wt p53 targets are biased towards gene activation. From these studies we identified and validated a number of new direct transcriptional targets of wt p53 including *DGKZ*, *FBXO22 *and *GDF9*.

The p53 mutants R175H, R249S, R273H, and R280K, when overexpressed in HME1 cells with a wt p53 background, showed no or highly compromised DNA binding relative to a comparable level of wt p53 alone. This supports a dominant negative effect of mt p53 on the wt p53 protein. There were very few changes in histone acetylation observed in cells overexpressing mt p53 with the most occurring in the R175H mutant. We observed no changes in DNA methylation in response to long-term expression of mt p53. In summary the mt p53 inhibited binding of wt p53 resulting in blocking of wt p53 epigenetic actions.

## Methods

### Cell culture

The cell lines hTERT-HME1 and MDA-MB-157 were purchased from the American Type Culture Collection (Rockville, MD, USA). The hTERT-HME1 cell lines harboring mt p53 genes were prepared in our lab previously and their cultivation was previously described [35]. The MDA-MB-157 cells were cultured as previously described [39]. Recombinant adenovirus serotype 5, containing wild-type p53 with a green fluorescent protein marker, was the kind gift of Bert Vogelstein and was propagated as previously described [39]. Adenovirus was added to hTERT-HME1 cells at 100 pfu/cell for 24 hours.

### Chromatin immunoprecipitation (ChIP)

A previously described ChIP protocol was used [48], except that the final purification of immunoprecipitated samples was done using a QIAquick^® ^PCR purification kit (Qiagen, Valencia, CA, USA) instead of phenol/chloroform extraction. p53 specific ChIP was performed using 2 μg of antibody clone DO-1 (Ab-6, #OP43, Calbiochem, EMD Chemicals, La Jolla, CA, USA) per 1.2 mL of diluted lysate from 10^7 ^cells. ChIP of acetylated histones H3 and H4 using antibodies against acetylated histones H3 (rabbit polyclonal IgG, #06-599, Upstate Biotech, Lake Placid, NY, USA) and H4 (rabbit polyclonal serum, #06-866, Upstate Biotech) were perfomed as previously described [39]. Immunoprecipitated DNA was quantified using PicoGreen dye (Invitrogen, Carlsbad, CA, USA) and BioTek FLx800 Multi-Detection Microplate Reader (BioTek Instruments, Winooski, VT, USA).

### Microarray detection of p53 binding, histone acetylation and DNA methylation

The human promoter microarray used in our study contains PCR fragments targeted to regions spanning 700 bp upstream and 200 bp downstream of the transcription start sites of 13,000 human genes. Primers for the microarray probes were obtained from the Whitehead Institute [37] and microarray preparation was described earlier [35].

For microarray analysis DNA was first amplified using the BioPrime DNA Labeling System (Invitrogen) with 1 mM dTTP used instead of labeled dUTP. Equal amounts (1 μg) of amplified ChIP and input DNA were labeled using the BioPrime DNA Labeling System with Cy3 or Cy5 dyes respectively using a double reaction per sample and 1/3 the recommended amount of dye. Cy3 and Cy5 labeled targets were mixed, then 20 μg of human Cot-1 DNA (Invitrogen) and 40 μg of yeast tRNA (Invitrogen) were added, and samples were dried under vacuum. Targets were dissolved in DMH-25 Domino Oligo Hybridization Buffer (The Gel Company, San Francisco, CA, USA), denatured and hybridized to processed microarray slides using an ArrayBooster (Advalytix, Munich, Germany) at 42°C for 16 h. Following hybridization, slides were washed with 2× SSC, 0.1% SDS for 5 min, then with 0.06× SSC, 0.1% SDS for 5 min, and finally with 0.06× SSC for 5 min, all at room temperature. Slides were scanned for Cy3 and Cy5 fluorescence using an Axon GenePix 4000 microarray reader (Axon Instruments, Inc., Foster City, CA, USA). Detailed amplification, labeling and hybridization protocols and microarray data are available in the ArrayExpress database: p53 binding [ArrayExpress:E-MEXP-1491], histone H3 acetylation [ArrayExpress:E-MEXP-1493] and histone H4 acetylation [ArrayExpress:E-MEXP-1494].

For 5-methylcytosine DNA analysis, DNA was immunoprecipitated using 2 μg/sample mouse monoclonal antibody 33 D3 (#AMM99021, Aviva Systems Biology, San Diego, CA, USA) specific for 5-methylcytosine DNA as previously described [49] and further analyzed on the promoter microarray. McrBC digestion analysis of DNA methylation and CpG island microarray hybridization was conducted as previously described [38].

### Statistical analysis of microarray data

The data from scanned microarray images were extracted using GenePix software and further analyzed using the limma package [50] for R [51]. p53 binding experiments were done in triplicate using dye swap for each replicate resulting in 6 hybridizations per cell line. Microarrays were normalized using loess function for within array normalization and quantile function for between array normalization. Finally, two linear model fits were calculated using inputs or MDA-MB-157 negative control ChIP samples as the common references, respectively. A list was generated of promoters that were enriched in the studied cell lines over both input and over MDA-MB-157 negative control at p-value ≤ 0.01. These promoters are referred to as bound by p53 within this paper. Acetylation of histones H3 and H4 and DNA methylation experiments were done in triplicate. A linear model fit was computed using input as a common reference. Contrasts of p53 over-expressing cell lines relative to control (parental and vector only transformed cells) were calculated using p-value ≤ 0.05. The significance of gene list overlaps was calculated using R as described previously [52].

### Real-time PCR

Equal amounts (1 ng) of p53 specific ChIP and input DNA were used for real-time PCR analysis. Primers were designed for potential p53 binding sites in promoter regions covered with probes on the promoter microarray for the genes *PLK3*, *FAS*, *APAF1*, *FBXO22*, *DDB2*, *DGKZ*, *MASPIN*, *MGC4771*, *SEMA3B*, *PCM1*, *GDF9*, *DPAGT1*, *SKI*, *SYK*, *CSPG2*, *OVOL1*, *PLXNB3*, *TSSC4*, *NR1H3*, *RPS27L*, *EVA1*, *ITPKB*, *ICT1*, *VSNL1*, *PRKAB2*, and *GAPDH*. Primers were designed for use with the Human Universal Probe Library Set (Roche Diagnostics, Indianapolis, IN, USA). Real-time PCR was conducted on an ABI Prism 7500 Sequence Detection System (Applied Biosystems, Foster City, CA, USA) using PerfeCta qPCR SuperMix, Low ROX (Quanta Biosciences, Gaithersburg, MD, USA) with a 95°C denaturation for 3 minutes followed by 45 cycles of 95°C for 15 seconds and 60°C for 45 seconds. Enrichment was calculated as previously described [39]. Primer sequences are available upon request.

### Real-time RT-PCR

Total RNA was isolated using the RNeasy^® ^Mini Kit (Qiagen). Reverse transcription was performed as previously described [39]. Primers were designed for *PLK3*, *FAS*, *APAF1*, *FBXO22*, *DDB2*, *DGKZ*, *MASPIN*, *MGC4771*, *SEMA3B*, *GDF9*, *SYK*, *CSPG2*, *PLXNB3*, *RPS27L*, *EVA1 *and *GAPDH *and used with the Human Universal Probe Library Set (Roche Diagnostics). PCR was run using cDNA generated from the equivalent of 15 ng of RNA per reaction as described above. All experiments were conducted in triplicate from three independent RNA isolations. Primer sequences are available upon request.

## Abbreviations

ChIP: chromatin immunoprecipitation; HME1: hTERT-immortalized human mammary epithelial cells; mt: mutant; wt: wild-type.

## Authors' contributions

LV carried out microarray analysis of p53 binding and histone acetylation, real time PCR, data analysis, figure preparation and drafted most of the manuscript. DJJ prepared cell lines stably overexpressing p53 mutants, performed real time RT-PCR and drafted the background section of the manuscript. PN carried out microarray analysis of DNA methylation. LV, DJJ and BWF designed the study and finalized the manuscript. All authors read and approved the final manuscript.

## Supplementary Material

Additional file 1 **List of p53 bound promoters**. File contains list of genes that were bound by p53 for all studies cell lines. Click here for file

Additional file 2 **List of differentially acetylated promoters**. File contains list of genes that had changed acetylation of histones H3 or H4 relative to control for all studies cell lines. Click here for file
